# Temporal genomic heterogeneity guiding individualized therapy in recurrent non-small cell lung cancer

**DOI:** 10.3389/fonc.2023.1116809

**Published:** 2023-07-12

**Authors:** Qiyu Fang, Xiaoying Wan, Angelica D’Aiello, Hui Sun, Weiquing Gu, Yixue Li, Caicun Zhou, Boxiong Xie, Qinfang Deng, Haiying Cheng, Songwen Zhou

**Affiliations:** ^1^ Medical College of Soochow University, Suzhou, Jiangsu, China; ^2^ Department of Oncology, Shanghai Pulmonary Hospital, Tongji University School of Medicine, Shanghai, China; ^3^ Department of Oncology, Montefiore Medical Center, Albert Einstein College of Medicine, Bronx, NY, United States; ^4^ Department of Thoracic Surgery, Shanghai Pulmonary Hospital, Tongji University School of Medicine, Shanghai, China; ^5^ School of Life Sciences and Biotechnology, Shanghai Jiao Tong University, Shanghai, China; ^6^ Chinese Academy of Sciences (CAS) Key Laboratory for Computational Biology, Chinese Academy of Sciences - Max-Planck-Gesellschaft (CAS-MPG) Partner Institute for Computational Biology, Shanghai Institute for Biological Sciences, Chinese Academy of Sciences, Shanghai, China

**Keywords:** dynamic gene sequencing, genotypic switch, temporal heterogeneity, EGFR, NSCLC

## Abstract

**Introduction:**

Despite the benefit of adjuvant systemic therapy for patients with resected non-small cell lung cancer (NSCLC), the risk of postoperative recurrence remains high. Our objective was to characterize temporal genetic heterogeneity between primary resected and recurrent tumors, and its impact on treatment outcomes.

**Methods:**

In this study, next-generation sequencing (NGS) testing was performed on tissue specimens and circulating tumor DNA (ctDNA) collected at postoperative recurrence, and results were compared to the genotypes of initial surgical specimens.

**Results:**

Of forty-five patients with matched primary and post-operative recurrent tumors, EGFR status switched in 17 patients (37.8%) at post-operative recurrence and 28 patients (62.2%) had no genotype change (17 mutant, 11 wild-type). Based on the changes of EGFR status, patients were divided into 4 groups. Following subsequent treatment with EGFR TKI o chemotherapy: In group A, with sustained sensitive mutation, the percentage achieving partial response (PR) was the highest, at 72.2%, the median progression-free survival (PFS) was 17 months, and the median overall survival (OS) was 44.0 months respectively; In group B, with genotype changed from wild-type to mutant, 50% achieved PR, PFS was 10 months, and OS was 35 months; In group C, in which mutant status shifted to wild-type or new co-mutation emerged, the percentage achieving PR was 30%, PFS was 9 months, and OS was 35 months. In group D, with sustained wild type, the percentage achieving PR was 27.3%, PFS was 8 months, and OS was 22 months.

**Discussion:**

Genotypic shift between paired primary and post-operative recurrent tumors was not infrequent, and this temporal genomic heterogeneity substantially impacted subsequent treatment outcomes.

## Introduction

Adjuvant therapy improves the survival of patients with resected non-small cell lung cancer (NSCLC), although 25–80% of patients experience postoperative recurrence (1, 2). Subsequent treatment strategy for patients with postoperative recurrences often depends on the genomic data determined from the initial surgical specimens. However, studies have shown that there is genomic temporal heterogeneity in recurrent and metastatic lung cancer after surgery. The coexistence of intratumoral driver gene heterogeneity and subcloning leads to clonal evolution, potentially resulting in genotypic changes and temporal heterogeneity (3). Lung cancer recurrence usually manifests as intrathoracic lesions or as extrapulmonary metastasis, such as in the brain or bone. Repeat genomic sequencing at recurrence or second primary has been used at some cases to guide treatment. At the same time, repeat tissue biopsy may not be feasible for some patients, so an important alternative is next-generation sequencing (NGS) of blood-derived circulating tumor DNA (ctDNA) (4).

In this study, NGS testing was performed on tissue specimens and ctDNA collected at postoperative recurrence, and results were compared to the genotypes of initial surgical specimens. The purpose of this study was to evaluate postoperative genotype changes and to identify the prevalence of clonal evolution in order to more accurately guide individualized treatment at recurrence.

## Materials and methods

### Patients

The study was conducted at the Department of Oncology at Shanghai Pulmonary Hospital from September 1, 2015 to April 1, 2018, including 45 NSCLC patients who experienced recurrence after surgery. The study was reviewed and approved by the institutional review boards, and in accordance with the Declaration of Helsinki and Good Clinical Practice. Informed consent was obtained for all subjects. The collected clinicopathological parameters included age, sex, smoking history, histology, TNM classification [based on the 8th edition of the American Joint Committee on Cancer (AJCC)/Union for International Cancer Control (UICC)], *EGFR* mutational status and types of subsequent treatment (including *EGFR*-TKIs). A person who had smoked <100 cigarettes in his or her lifetime was deemed as a never-smoker.

### Sample collection and processing

Initial surgical specimens and tissue and blood samples, and those at recurrence, were collected. Tumor content on hematoxylin and eosin-stained slides were assessed by board-certified pathologists for all formalin-fixed, paraffin-embedded tissue specimens. Tissue specimens were deemed evaluable if tumor cells were identified. Eight sections of FFPE samples 5-10 μm were extracted using the QIamp DNA FFPE Tissue Kit (Qiagen, Hilden, Germany) according to the manufacturer’s instructions. Ten millimeters of fasting peripheral blood was collected into Streck blood collection tubes (BCT; Streck, Omaha, NE, US). Samples were transported at room temperature and processed at Shanghai Smartquerier Biomedicine Co. Ltd within 48 hours according to Streck BCT protocol. Samples were centrifuged at 2500g for 10 minutes. The supernatant serum was then collected and centrifuged at 16,000g for an additional 10 minutes. The resultant 3-5mL of supernatant was collected and circulating tumor DNA (ctDNA) was extracted using a QIAamp Circulating Nucleic Acid Kit (Qiagen, Hilden, Germany). Germline DNA was extracted from the supernatant after the first centrifugation using a QIAamp DNA Blood Mini Kit (Qiagen, Hilden, Germany). NGS was used to detect gene sequencing in surgical specimens and in plasma at recurrence. Sequencing of ctDNA by NGS was performed according to a previously published study to identify genetic mutations (5). A plasma and germline DNA library was used for NGS. The list of 156 genes is provided in **Supplementary Table 1**.

### Data collection

The median follow up period was thirty-seven months. No second primary cancers were found at the end of follow-up. All patients had physical examinations monthly, as well as chest CT scans, abdominal color Doppler ultrasound, bone scan, and brain MRI at 3-month intervals. Tumor response were determined by Response Evaluation Criteria in Solid Tumors (RECIST)1.1 criteria as complete response (CR), partial response (PR), stable disease (SD), or progressive disease (PD). Disease control rate (DCR) was defined as the proportion of cases with complete, partial response, or stable disease. The objective response rate (ORR) was defined as the proportion of patients with complete or partial response. The endpoint of the study was progression-free survival (PFS). Recurrent progression-fee survival (rPFS) was calculated from the date of the treatment after recurrence (1^st^ *EGFR*-TKI treatment or chemotherapy) until progressive disease (PD) or death due to any cause. Recurrent overall survival OS (rOS) was defined as the time of recurrence to death from any cause. Patients with *EGFR*-sensitive mutations were treated with *EGFR*-TKI as first-line treatment. The *EGFR*-TKIs used included gefitinib (250 mg, once a day), erlotinib (150 mg, once a day) and icotinib (125 mg, three times a day). Platinum-based doublet chemotherapy was given to patients without driver mutations at recurrence.

### Statistics

The categorical variables were compared with the Chi-square test or Fisher exact test when expected count in each category were less than 5. Survival analysis was determined by Kaplan-Meier curves with two-sided log rank tests. The Cox proportional hazards model with calculated hazard ratios (HR) and 95% confidence interval (CI) was applied to adjust for potential confounders. Statistical significance was defined as Two-sided P < 0.05. All statistical analyses were performed and displayed using SPSS statistical software, version 22.0 (SPSS Inc., Chicago, IL, USA) and GraphPad Prism 9.0.0.

## Results

### Clinical characteristics

Of the 45 patients, 17 (37.8%) were female and 28 (62.2%) were male. The median age at post-operative recurrence was 58 years (range 43-79 years. There were 43 cases of adenocarcinoma nd 2 case of squamous cell carcinoma. Patients had stages ranging from IB to IIIA. Thirteen of the total patients with more advanced disease received 4-cycles of adjuvant platinum-based doublet chemotherapy, and the majority of patients with N2 disease received mediastinal radiotherapy after operation. Twenty-two patients developed postoperative recurrence in the lungs, while 12 patients had disease after recurrence in the bone, and 11 patients had local recurrence in mediastinal lymph nodes. None of the patients had adjuvant targeted therapy (**Table 1**).

**Table 1:** Baseline clinical characteristics of Chinese patients with non-small cell lung cancer (n=45).

Chacteristic		Number (%)
Gender	Male	28 (62.2)
	Female	17 (37.8)
Smoking history	Never smoker	27 (60.0)
	Smoker	18 (40.)
Family history		2 (4.4)
TNM Stage	IA	13 (28.9)
	IB	11 (24.5)
	IIA	5 (11.1)
	IIB	2 (4.4)
	IIIA	14 (31.1)
Histopathology	Adenocarcinoma	43 (95.6)
	Squamous cell carcinoma	2 (4.4)
Site of post-operative recurrence	Pleura	9 (20.)
	Lung	22 (48.9)
	Bone	12 (26.7)
	Brain	8 (17.8)
	Liver/spleen	2 (4.4)
	Mediastinal lymph node	11 (24.4)

### Shifts of EGFR mutation status at recurrence

**Figure 1:**
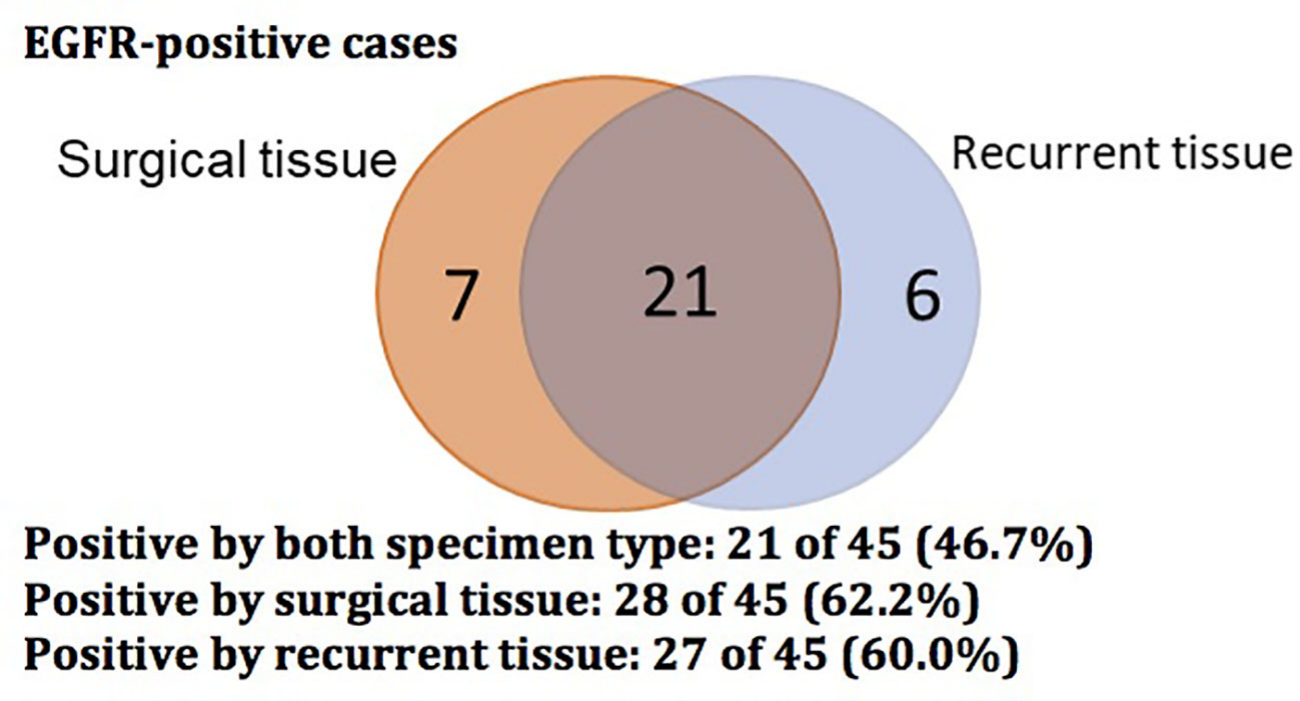
Venn diagram showing EGFR-positive status of 45 cases with surgical and recurrent specimens.

The *EGFR* mutational status changed in 17 patients (37.8%) at post-operative recurrence. The rest of 28 patients (62.2%) had no change (17 mutations, 11 wild-type; **Tables 2**, **3**; **Figure 1**). We divided these cases into 4 groups by comparing results of sequencing matched primary and post-operative recurrent NSCLC tumors (**Table 3**):

**Table 2:** Alteration of EGFR Mutation Status.

	Surgical Specimen	
Recurrence	Mutant (N, %)	Wild type (N, %)
Mutant	21 (46.7)	6 (13.3)
Wildtype	7 (15.5)	11 (24.5)

**Table 3:** Transformation of genome.

	No.	Gender	Age	pTNM	Surgical tissue	Postoperative adjuvant therapy	Site(s) of recurrence	Recurrence specimen		Current treatment
								Tissue	Blood	
Wild type→Mutant	1	Female	61	IIB	Wild type	Chemotherapy	Bilateral lung Mediastinal lymph node Pleura	*EGFR* exon21 p.L858R	*EGFR* exon21 p.L858R	Gefitinib
	2	Male	53	IA	Wild type	None	Bone		*EGFR* exon21 L858R	Gefitinib
	3	Female	53	IIA	Wild type	Chemotherapy	Bilateral lung Mediastinal lymph node Brain	*EGFR* exon 21 p.L858R	*EGFR* exon 21 p.T854A	Gefitinib
	4	Male	52	IIIA	Wild type	Chemoradiotherapy	Brain		*EGFR* exon19 del	Gefitinib
	5	Female	70	IIIA	Wild type	Chemoradiotherapy	Bone Bilateral lung	*EGFR* exon 21 p.L858R	*EGFR* exon 21 p.L858R	Gefitinib
	6	Male	43	IB	Wild type	Chemotherapy	Pleural Lung	*EGFR* exon19 del	*EGFR* exon19 del	Icotinib
Mutant→Wild type	7	Male	70	IA	*EGFR* exon19 del	None	Pleura	*EGFR p.A767delinsAQRG*	*EGFR p.A767delinsAQRG*	Gefitinb
	8	Female	48	IA	*EGFR* exon19 del	None	Bilateral lung	Wild type	Wild type	Icotinib
	9	Male	58	IIB	*EGFR* exon19 del	Chemotherapy	Bilateral lung	Wild type	Wild type	Gefitinib
	10	Female	54	IIA	*EGFR* exon19 del	Chemoradiotherapy	Mediastinal lymph node	Wild type	Wild type	Gefitinib
	11	Male	56	IA	*EGFR* exon19 del	None	Bone		Wild type	Gefitinbi
	12	Male	50	IA	*EGFR* exon 21 p.L858R	None	Pleura	Wild type	Wild type	Gefitinbi
	13	Male	67	IIIA	*EGFR* exon19 del	Chemoradiotherapy	Brain		Wild type	Icotinib
EGFR Co-Mutation	14	Male	56	IIA	*EGFR* exon19 del	Chemotherapy	Mediastinal lymph node Bilateral Lung Liver Spleen	*EGFR* exon19 del *TP53* p.R273C exon8	*EGFR* exon19 del *TP53* p.R273C exon8	Gefitinib
	15	Female	53	IA	*EGFR* exon19 del	None	Bilateral lung Bone	*EGFR* exon19 del *TP53* exon10 p.R342	*EGFR* exon19 del *TP53* exon10 p.R342	Icotinib
	16	Male	54	IB	*EGFR* exon 21 p.L858R	Chemotherapy	Pleura	*EGFR* p.L858R exon21 *PIK3CA* p.E542K exon10	*EGFR* p.L858R exon21 *PIK3CA* p.E542K exon10	Gefitinib
Mutation consistent	17	Male	79	IB	*EGFR* exon19 del	Chemotherapy	Bilateral lung	*EGFR* exon21 p.L858R	*EGFR* exon21 p.L858R	Icotinib
	18	Male	55	IA	*EGFR* exon19 del	None	Bilateral lung	*EGFR* exon19 del	*EGFR* exon19 del	Gefitinib
	19	Male	59	IA	*EGFR* exon19 del	None	Mediastinal lymph node	*EGFR* exon19 del	*EGFR* exon19 del	Gefitinib
	20	Male	63	IB	*EGFR* exon19 del	Chemotherapy	Bone Bilateral lung	*EGFR* exon19 del	*EGFR* exon19 del	Ictonitb
	21	Male	60	IB	*EGFR* exon19 del	Chemotherapy	Bilateral lung	*EGFR* exon19 del	*EGFR* exon19 del	Icotinib
	22	Male	72	IB	*EGFR* exon 21 p.L858R	Chemotherapy	Mediastinal lymph node Bilateral lung	*EGFR* exon 21 p.L858R	*EGFR* exon 21 p.L858R	Gefitinib
	23	Female	50	IIIA	*EGFR* exon19 del	Chemoradiothearpy	Bilateral lung	*EGFR* exon19 del	*EGFR* exon19 del	Gefitinib
	24	Female	58	IIIA	*EGFR* exon19 del	Chemoradiotherapy	Mediastinal lymph node	*EGFR* exon19 del	*EGFR* exon19 del	Erlotinib
	25	Male	46	IIIA	*EGFR* exon19 del	Chemoradiotherapy	Bilateral lung	*EGFR* exon19 del	*EGFR* exon19 del	Gefitinib
	26	Male	49	IIIA	*EGFR* exon19 del	Chemoradiothearpy	Bone		*EGFR* exon19 del	Gefinitib
	27	Female	62	IIA	*EGFR* exon 21 p.L858R	Chemotherapy	Bone		*APOB* exon29 p.I4381L,*ARAF* exon7 p.R188H, *SPTA1* exon37 p.E1761G	Gefitinib
	28	Female	59	IB	*EGFR* exon19 del	Chemotherapy	Pleural	*EGFR* exon19 del	*EGFR* exon19 del	
	29	Female	60	IA	*EGFR* exon19 del	None	Bone		*EGFR* exon19 del	Icotinib
	30	Female	55	IA	*EGFR* exon 21 p.L858R	None	Bone Bilateral lung	*EGFR* exon 21 p.L858R	*EGFR* exon 21 p.L858R	Gefitinib
	31	Female	61	IA	*EGFR* exon19 del	None	Bilateral lung	*EGFR* exon19 del	*EGFR* exon19 del	Icotinib
	32	Male	62	IIIA	*EGFR* exon 21 p.L858R	Chemoradiotherapy	Pleural	*EGFR* exon 21 p.L858R	*EGFR* exon 21 p.L858R	Erlotinbi
	33	Male	56	IIIA	*EGFR* exon19 del	Chemoradiotherapy	Mediastinal lymph node Bilateral lung	*EGFR* exon19 del	*EGFR* exon19 del	Icotinib
	34	Female	60	IIIA	*EGFR* exon 21 p.L858R		Pleural Bone	*EGFR* exon 21 p.L858R	*EGFR* exon 21 p.L858R	Gefitinib
Wild type consistent	35	Male	55	IIA	Wild type	Chemoradiotherapy	Bone Liver	Wild type	Wild type	Chemotherapy
	36	Male	55	IIIA	Wild type	Chemoradiotherapy	Bilateral lung	Wild type	Wild type	Chemotherapy
	37	Male	44	IIIA	Wild type	Chemoradiotherapy	Brain		Wild type	Chemotherapy
	38	Male	57	IB	Wild type	Chemotherapy	Brain		Wild type	Chemotherapy
	39	Male	64	IB	Wild type	Chemotherapy	Bilateral lung Mediastinal lymph node	Wild type	Wild type	Chemotherapy
	40	Female	60	IB	Wild type	Chemothearpy	Bilateral lung Mediastinal lymph node	Wild type	Wild type	Chemotherapy
	41	Male	66	IIA	Wild type	Chemotherapy	Bilateral lung Mediastinal lymph node	Wild type	Wild type	Chemotherapy
	42	Male	66	IIA	Wild type	Chemotherapy	Bone		Wild type	Chemotherapy
	43	Male	74	IA	Wild type	None	Pleural	Wild type, *NRAS* p.A146T	Wild type, *NRAS* p.A146T	Chemotherapy
	44	Male	60	IB	Wild type	Chemothearpy	Brain		Wild type	Chemotherapy
	45	Female	54	IA	Wild type	None	Brain		Wild type	Chemotherapy

Group A (18 cases, 40%): sustained sensitive E*GFR* mutations in both primary and recurrent tumors. Group B (6 cases, 13.3%): *EGFR* wild type in the primary tumors but *EGFR* sensitive mutation in the recurrent tumors.

Group C (10 cases, 22.2%): 7 cases had *EGFR* mutations in the primary tumors but wild type at recurrence, of which, 1 case had *EGFR* p.A767delinsAQRG, and 3 patients developed an *EGFR*-co-mutation in the recurrent specimens: 1) *EGFR* exon19 del and TP53 p.R273C exon8; 2) *EGFR*exon19 del and *TP53* exon10 p.R342, and 3) *EGFR* p.L858R exon21 and *PIK3CA* p.E542K exon10.

Group D (11 cases, 24.4%): Sustained *EGFR* wild-type in both primary and recurrent specimens.

### Clinical responses in groups with different EGFR mutation shift

At recurrence, patients with a sensitive *EGFR* mutation were treated with first-line *EGFR*-TKI, and those in the wild-type group received chemotherapy. Twenty-two patients had partial response, 21 patients had stable disease, and 2 cases showed disease progression.

**Figure 2:**
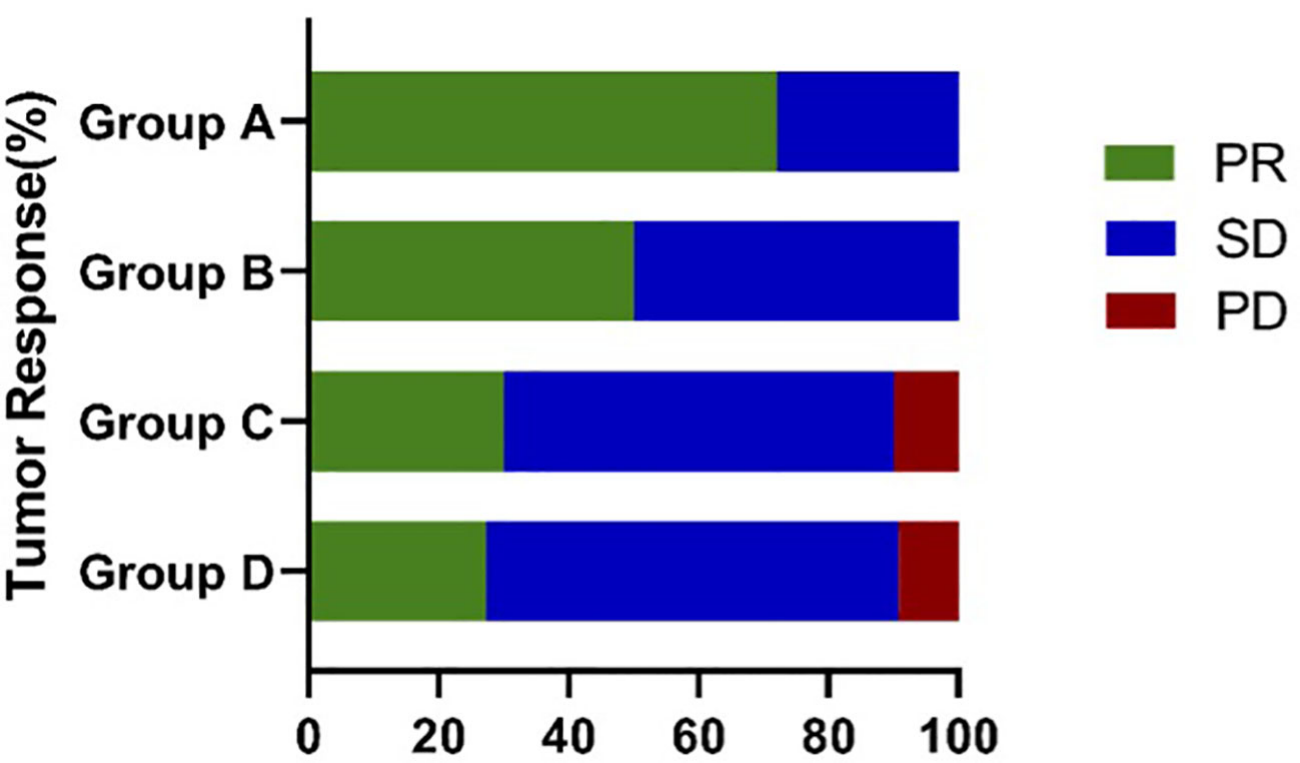
Response outcome between different groups.

After receiving the first-line treatment, the disease control rates were high: 100% (Group A), 100% (Group B), 90% (Group C), and 90.0% (Group D). The highest PR rate of 72.2% was observed in Group A with sustained sensitive EGFR mutations and received EGFR TKIs. Patients in group B had a PR rate of 50%. In group C, the PR rate was 30%. In group D with sustained EGFR wild-type, the PR rate with chemotherapy was 27.3% (**Table 4**; **Figure 2**). Likely due to small sample size, there was no significant difference in PR rate among the four groups (χ2 = 7.273, P=0.061; **Table 5**), but patients in group A had higher PR rates than those in group D 185 (χ2 = 5.730, P=0.027).

**Table 4:** Tumor response of first-line EGFR-TKI or chemotherapy in patients.

		Tumor Response						Total No.
		Partial Response		Stable Disease		Progressive Disease		
Group	Treatment	No.	%	No.	%	No.	%	
A	EGFR-TKI	13	72.2	5	27.8	0	0.0	18
B	EGFR-TKI	3	50.0	3	50.0	0	0.0	6
C	EGFR-TKI	3	30.0	6	60.0	1	10.0	10
D	Chemotherapy	3	27.3	7	63.6	1	9.1	11
Total		22	48.9	21	46.7	2	4.4	45

**Table 5:** PR rate among groups.

Groups	χ2	*P*
A group vs B group	0.965	0.362
A group vs C group	4.755	0.050
A group vs D group	5.730	0.027
B group vs C group	0.635	0.607
B group vs D group	0.866	0.600
C group vs D group	0.019	1.000

### Management of brain metastases at time of recurrence

Most patients with brain metastases at recurrence had mild symptoms only. Patients with *EGFR* mutations and brain metastases detected at time of recurrence were started on EGFR targeted therapy and had repeat MRI brain at 1 month following treatment initiation. Patients did not receive brain radiotherapy if the brain metastases had significantly improved following one month of EGFR TKI therapy. Wild type patients with brain metastases were treated with radiation therapy in addition to chemotherapy if symptomatic.

### Survival outcomes in groups with different EGFR mutation shift

**Figure 3:**
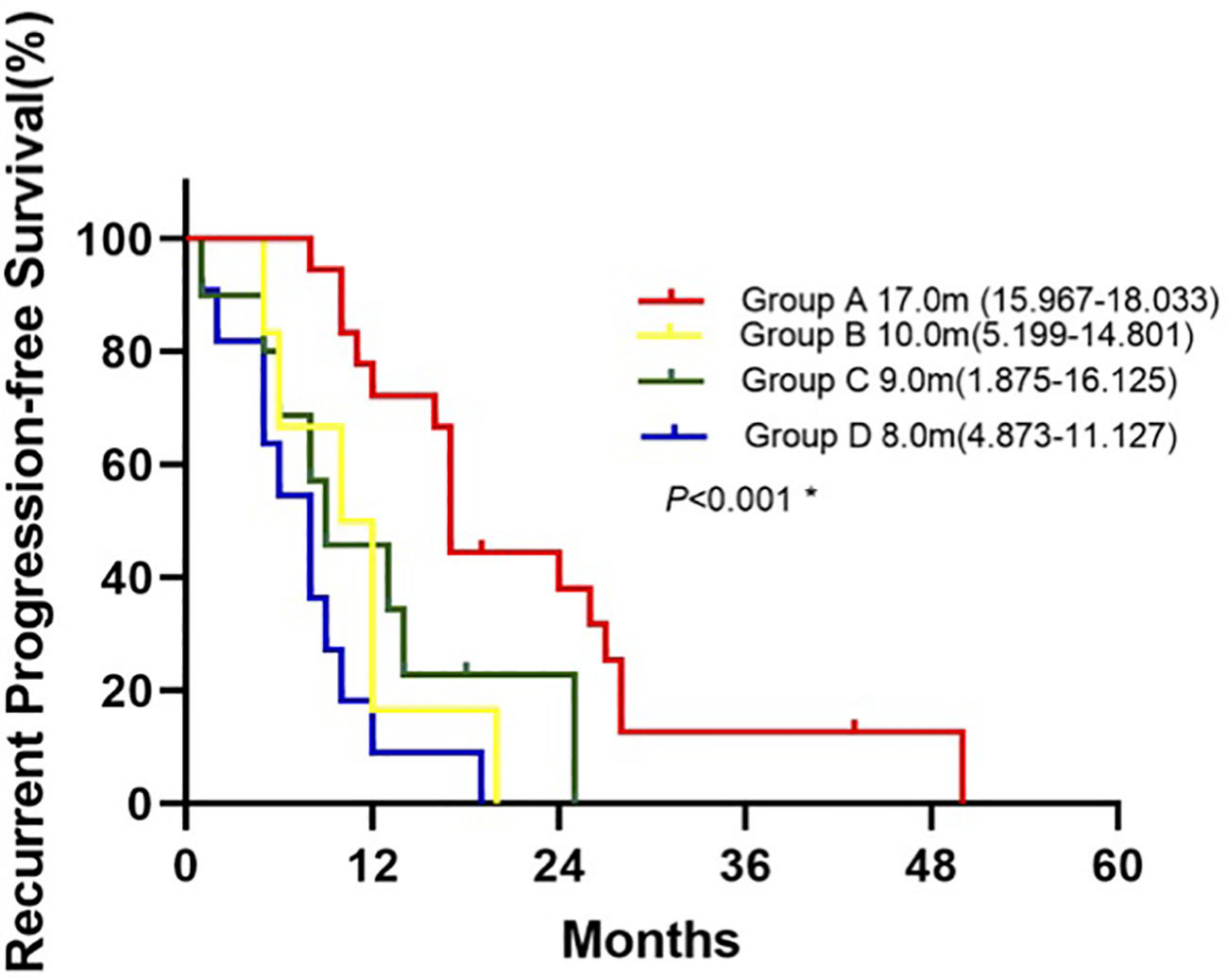
Kaplan-Meier curve for recurrent progression-free survival of patients between groups.*Log-rank test: Group A vs. Group B(P=0.008), Group A vs. Group C (P=0.016), Group A vs. Group D(P<0.001), Group B vs. Group C(P=0.620), Group B vs. Group D(P=0.163), Group C vs. Group D(P=0.156).

Patients in Group A had the longest median rPFS of the four groups at 17.0 months [95% CI 15.967-18.033], compared to 10.0 months with group B [95% CI 5.199-14.801], 9.0 months with group C [95% CI 1.875-16.125], and 8.0 months with group D [95% CI 4.873-11.127]. The difference in rPFS was statistically significant between patients in Group A and Group B(P=0.008), Group A and Group C (P=0.016), Group A and Group D (P = 0.001) (**Figure 3**). Univariate analysis identified mutations in both surgical and recurrent specimens as being significantly associated with better PFS (HR 0.172, 95% CI 0.071-0.421; P <0.001) (**Table 6**). After adjusting for confounding factors, the multivariate analysis showed that sustained *EGFR* mutations remained as predictors of better PFS (HR 0.163, CI 0.064-0.415; P <0.001).

**Table 6:** Predictors on prognostic factors in Cox Regression Analysis for 45 NSCLC Patients.

	Univariate analysis			Multivariate analysis		
	HR	95% CI	*P*	HR	95% CI	*P*
rPFS						
Gender						
Female	1.000			1.000		
Male	1.320	0.690-2.526	0.402	0.981	0.402-2.393	0.967
Smoking history						
NO	1.000			1.000		
YES	1.570	0.833-2.958	0.163	1.404	0.577-3.416	0.454
Age Group						
Group D	1.000					
Group A	0.172	0.071-0.421	0.000	0.163	0.064-0.415	0.000
Group B	0.583	0.213-1.595	0.293	0.535	0.182-1.567	0.254
Group C	0.491	0.194-1.243	0.133	0.515	0.201-1.321	0.167
rOS						
Gender						
Female	1.000			1.000		
Male	1.497	0.665-3.367	0.329	1.396	0.451-4.321	0.563
Smoking history						
NO	1.000			1.000		
YES	1.499	0.691-3.249	0.305	0.895	0.289-2.773	0.848
Age Group						
Group D	1.000			1.000		
Group A	0.368	0.141-0.961	0.041	0.361	0.131-1.000	0.050
Group B	0.693	0.212-2.267	0.544	0.817	0.236-2.833	0.750
Group C	0.463	0.154-1.388	0.169	0.556	0.170-1.821	0.332

**Figure 4:**
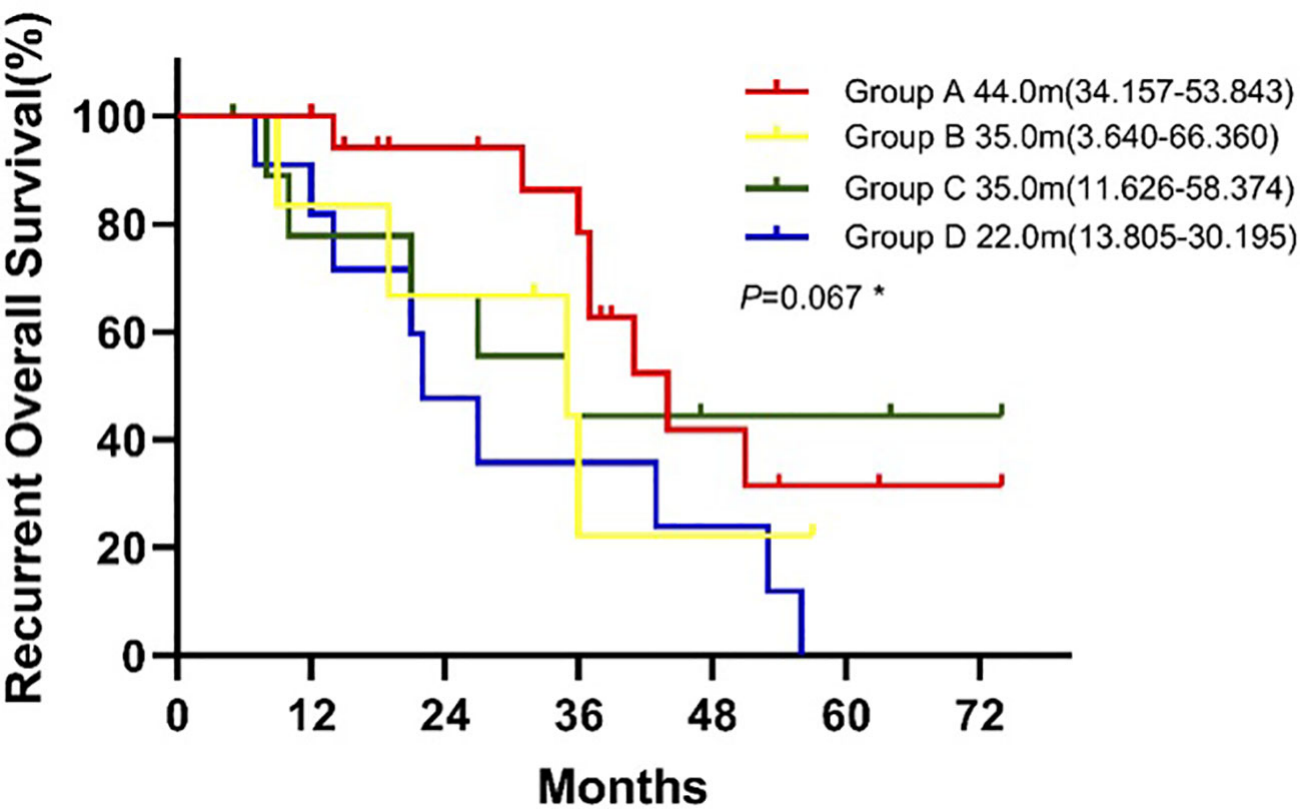
Kaplan-Meier curve for recurrent overall survival of patients between groups. * Log-rank test: Group A vs. Group B(P=0.171), Group A vs. Group C (P=0.641), Group A vs. Group D(P=0.039), Group B vs. Group C(P=0.715), Group B vs. Group D(P=0.482), Group C vs. Group D(P=0.182).

The median rOS in four groups were 44.0 months (95% CI 34.157-53.843 for group A, 35.0 months (95% CI, 3.640-66.360) for group B, 35.0 months (95% CI 11.626-58.374) for group C, and 22.0 months (95% 13.805-30.195) for group D. Patients in group A had a significantlyy longer OS than patients in group D (P = 0.039; **Figure 4**). Compared with group D, univariate analysis showed mutations in both surgical and recurrent specimens as being significantly associated with favorable OS (HR 0.368, 95% CI 0.141-0.961; P=0.041).

## Discussion

Tumor heterogeneity, with genetic and non-genetic diversity, is a key feature of cancer (3). Spatial heterogeneity may be observed in different parts of a given tumor, and temporal heterogeneity may occur with recurrent or metastatic disease. Tumor heterogeneity may result from distinct clonal evolution, mutational processes, and selection pressures. NSCLC, especially lung adenocarcinoma, is one of the most heterogeneous cancers. Drugs, environment, and other factors promote dynamic, competitive evolution of tumor subclones, and dominant clones arise by survival of the fittest (6). Failure to recognize and detect tumor heterogeneity may lead to treatment failure in lung cancer (7). Therefore, it is important to monitor the dynamics of oncogenic driver mutations to guide the treatment of lung cancer in an individualized and precise manner.

Activating somatic mutations in the *EGFR* receptor tyrosine kinase are the most common targetable driver alterations identified in NSCLC, occurring in up to 16% patients with adenocarcinoma (8). *EGFR* belongs to a family of receptor tyrosine kinases known as ERBB. Under physiologic conditions, *EGFR* activates multiple downstream signaling pathways including mitogen-activated protein kinases (MAPK), PI3K/AKT/mTOR, and JAK/STAT leading to cellular proliferation and oncogenesis (9). There are three generations of *EGFR* TKIs currently in clinical practice with varying mechanisms of action and efficacy for particular mutation subtypes (10). First generation *EGFR* TKIs, including gefitinib and erlotinib, reversibly compete with ATP for binding to the EGFR tyrosine kinase receptor preventing downstream signaling. Second generation *EGFR* TKIs including neratinib, dacomitinib and afatinib irreversibly bind to the *EGFR* receptor tyrosine kinase domain and target other members of the ERBB family. While NSCLC with EGFR exon 19 deletions of exon 21 L858R mutations, those harboring the T90M mutation are associated with resistance. The third generation *EGFR* TKIs such as osimertinib form an irreversible covalent bond with the cysteine-797 residue in the *EGFR* ATP binding site, with potent activity against the *EGFR*-T790M mutation. Identification of *EGFR* mutations and development of selective *EGFR* inhibitors has revolutionized the management of NSCLC for a molecularly-defined group of patients leading to improved clinical outcomes (11). In addition, previous studies implicated that *EGFR* mutation status may differ between paired primary and metastatic NSCLC tumors in a portion of patients. A meta-analysis found that the discordance rate was 14.5% (12), and another study reported a discordance rate of 5%–17% (13). These results indicate that primary or metastatic samples alone are insufficient to reflect genomic features due to temporal heterogeneity.

In our study, we also found discordance between the primary tumor at initial resection and at the time of recurrence. Genotypic changes were observed in 17 patients (37.8%) at post-operative recurrence. Six patients had a mutation from wild type, 7 patients changed to wild type from mutant, 3 patients had developed new *EGFR*-co-mutation in recurrent specimens, and 1 patient had a sensitive mutation with changed from 19del to L858R. Twenty-eight patients (62.2%) had no change in genotype (17 mutations, 11 wild-type). One possible explanation for the observed genotypic shift from *EGFR*-mutant to wild type might be due to spatial heterogeneity of the tumor, which could result in an uneven distribution of genetic subpopulations within a single tumor or across disease sites. Another possibility is the potential for a false negative result, which could exist due to low tumor load or inadequate tissue sampling. Collection of additional tissue specimens or blood samples for repeated NGS testing would be one means to more effectively rule this out. As no patients received adjuvant *EGFR* TKIs after surgical resection, we would not expect the change in mutation status to be related to targeted therapy. Of note, our discordance rate was higher than the ones in previous reports, which may be due to the use of ctDNA analysis at time of recurrence in our study. At disease progression, insufficient material obtained by tissue biopsy may preclude complete sequencing, and this may occur in up to 20–25% of needle biopsies (14, 15). In this case, ctDNA analysis can provide otherwise complimentary sequencing data that could significantly impact treatment decisions.

Importantly, given the potential for tumor heterogeneity, diverse genetic alterations leading to drug resistance may be detected by ctDNA analysis across different metastatic sites that may not be detected by biopsy of a single site (16). Using ctDNA analysis, Paweletz et al. found two mutations (exon19 of *EGFR*, high levels of *MET* amplification), which was not previously detected in tumor tissue (17). Previous work also compared mutations found in plasma and in tumor samples from NSCLC patients, and additional mutations were found in ctDNA analysis in several genes: *EGFR*, *KRAS, PIK3CA*, and *TP53* (18). Another study showed that only 1 of 9 patients had a plasma ctDNA mutational profile that was completely consistent with the mutational profile of the biopsied tumor (19). Nevertheless, there is limitation to characterize tumor heterogeneity using tumor biopsy tissues, whereas plasma ctDNA analysis appears to be a powerful tool to monitor the dynamic changes or heterogeneity in lung cancer. One advantage of ctDNA compared to tissue based NGS is its potential for overcoming the spatial heterogeneity that could exist within a particular tumor, or across various metastatic sites. As a result, ctDNA may characterize the genomic profile of a tumor more comprehensively than a single tissue biopsy alone.

In addition to detecting genetic changes, the dynamic monitoring of mutational status and tumor heterogeneity with ctDNA analysis also predicted response to therapy and helped to guide personalized treatment. Group A, in which there were sensitizing *EGFR* mutations at initial resection and at recurrence, had the highest PR rate of 72.2%, with a median rPFS of 17.0 months and rOS of 41.0 months. Patients from group B were newly found to have sensitizing *EGFR* mutations and gained access to *EGFR*-TKI therapy at recurrence; These patients had a PR rate of 50%, rPFS of 10 months, and rOS of 35.0 months, which appeared to be numerically inferior to group A and tumor heterogeneity may play a role. Group C, which shifted from having a sensitizing *EGFR* mutation to wild-type, achieved PR in 30%, rPFS of 9 months, and rOS of 35.0 months. In group D, patients received chemotherapy, the PR rate was only 27.3%, with rPFS of 8 months, and rOS of 22.0 months. Overall, patients in group A had significantly better rPFS, rOS and a higher PR rate than those in group D. For patients treated with *EGFR*-TKI, patients of group A owned longer rPFS than group B and group C, that indicated the low heterogeneity of tumors may associated with better survival although there was no significant difference in survival among other groups, possibly due to small sample size.

NGS analysis can help to identify resistance mechanisms to *EGFR*-TKI *(* 20, 21), such as certain *EGFR* co-mutations. In such patients harboring *EGFR* co-mutations, the response to *EGFR*-targeted therapy was reported to be significantly lower, and survival appeared shorter (22–24). In group C, we found a *TP53* co-mutation in 2 patients. Mutated *TP53* not only loses its tumor suppressor function but also are often associated with poor prognosis (25), regardless of *EGFR* status. In one study of 43 patients with *TP53* mutations, PFS with first-line *EGFR*-TKI was significantly shorter compared to those with wild type TP53, but OS was not significantly different (26). The survival of patients taking first-line *EGFR*-TKI with *EGFR* and *TP53* co-mutations, were shorter than those with only *EGFR* mutations (PFS 4.2 months vs. 12.5 months and OS 16.2 months vs. 32.3 months) (27). In our study, one patient whose *TP53* p.R273C exon 8 allele frequency was 32.6% had taken first-line *EGFR*-TKI for only 5 months before disease progression and had an OS of 10 months. Another patient had *TP53* exon10 p.R342 with an allele frequency of 5.7% and achieved PFS of 8 months.

*PIK3CA* alterations are also associated with poor prognosis in NSCLC, with significantly shorter PFS and OS compared to wild type (28). Alterations in *PIK3CA* are associated with both acquired and primary resistance to EGFR-TKIs in 1–3% of patients (28). One patient in our study had a *PIK3CA* p.E542K exon 10 alteration (allele frequency 0.155%) and was treated with first-line *EGFR* TKI, but had disease progression after 6 months, with an OS of 21 months. In another case, *EGFR* exon 19 del was no longer detectable but *EGFR* P. A767 delins AQRG (allele frequency 5.0%) was found by ctDNA analysis at the time of post-operative recurrence. The patient had PD just one month after initiating *EGFR* TKI, with OS of 8 months.

Genetic sequencing of the initial tumor specimen is often used to determine therapy at recurrence although tumor spatial heterogeneity may lead to poorer efficacy to targeted therapy (29, 30). In our study, through the analysis of matched primary and post-operative recurrence in 45 patients, we observed two key findings: first, temporal genetic heterogeneity occurred relatively commonly between the primary and post-operative recurrent NSCLC tumors; Second, temporal heterogeneity might influence the therapeutic efficacy of first-line EGFR TKI treatment at relapse. Therefore, the genetic sequencing of surgical specimens should not be used as the sole guide for targeted therapy either at initial diagnosis or recurrence. The addition of ctDNA analysis, which is noninvasive and sensitive, can be used to assess tumor heterogeneity and to guide personalized treatment. In our study, ctDNA results were not available for patients at time of initial resection as ctDNA was not widely used (years 2013-2016); however, it would be interesting to study how ctDNA results at recurrence would compare to those obtained at time of initial resection.

## Conclusion

In this study, through dynamic analysis of matched primary and post-operative recurrence in 45 patients, we found that genotypic shift was not infrequent in the relapsed tumors, and this temporal genomic heterogeneity substantially impacted subsequent treatment outcomes. Our study suggests that dynamic evaluation of genomic profile, especially oncogenic drivers such as *EGFR* mutational status, at cancer recurrence or relapse (or second primary) is warranted to tailor subsequent individualized therapy.

## Data availability statement

The data analyzed in this study is subject to the following licenses/restrictions: Data not available due to ethical restrictions. Requests to access these datasets should be directed to songwenzhou2017@vip.126.com.

## Ethics statement

The studies involving human participants were reviewed and approved by Shanghai Pulmonary Hospital. Written informed consent for participation was not required for this study in accordance with the national legislation and the institutional requirements.

## Author contributions

Study conception and design: SZ, CZ, BX, YL. Acquisition of data: QF, XW, QD and HS. Analysis and interpretation of data: QF, XW, QD, HS,WG, AD, EL, and HC. Drafting and revising: QF, XW, QD, SZ, CZ, BX, AD, EL, HC and YL. Approving final version of manuscript: All authors. SZ, CZ, BX, YL take responsibility for the integrity of the data analysis. All authors contributed to the article and approved the submitted version.

## References

[1] van den Berg LL Klinkenberg TJ Groen HJM Widder J. Patterns of recurrence and survival after surgery or stereotactic radiotherapy for early stage NSCLC. J Thorac Oncol (2015) 10(5):826–31. doi: 10.1097/JTO.0000000000000483 25629639

[2] Yamazaki K Sugio K Yamanaka T Hirai F Osoegawa A Tagawa T. Prognostic factors in non-small cell lung cancer patients with postoperative recurrence following third-generation chemotherapy. Anticancer Res (2010) 30(4):1311–5. 20530445

[3] Ramón Y Cajal S Sesé M Capdevila C Aasen T De Mattos-Arruda L Diaz-Cano SJ. Clinical implications of intratumor heterogeneity: challenges and opportunities. J Mol Med (Berl) (2020) 98(2):161–77. doi: 10.1007/s00109-020-01874-2 PMC7007907 31970428

[4] Rolfo C Mack P Scagliotti GV Aggarwal C Arcila ME Barlesi F. Liquid biopsy for advanced NSCLC: a consensus statement from the international association for the study of lung cancer. J Thorac Oncol (2021) 16(10):1647–62. doi: 10.1016/j.jtho.2021.06.017 34246791

[5] Deng Q Fang Q Sun H Singh AP Alexander M Li S. Detection of plasma EGFR mutations for personalized treatment of lung cancer patients without pathologic diagnosis. Cancer Med (2020) 9(6):2085–95. doi: 10.1002/cam4.2869 PMC7064093 31991049

[6] Amirouchene-Angelozzi N Swanton C Bardelli A. Tumor evolution as a therapeutic target. Cancer Discov (2017) 7:805–17. doi: 10.1158/2159-8290.CD-17-0343 28729406

[7] Yap TA Gerlinger M Futreal PA Pusztai L Swanton C. Intratumor heterogeneity: seeing the wood for the trees. Sci Transl Med (2012) 4(127):127ps10. doi: 10.1126/scitranslmed.3003854 22461637

[8] Rosell R Moran T Queralt C Porta R Cardenal F Camps C. Screening for epidermal growth factor receptor mutations in lung cancer. N Engl J Med (2009) 361(10):958–67. doi: 10.1056/NEJMoa0904554 19692684

[9] Roskoski R. The ErbB/HER family of protein-tyrosine kinases and cancer. Pharmacol Res (2014) 79:34–74. doi: 10.1016/j.phrs.2013.11.002 24269963

[10] Chhouri H Alexandre D Grumolato L. Mechanisms of acquired resistance and tolerance to EGFR targeted therapy in non-small cell lung cancer. Cancers (Basel) (2023) 15(2):504. doi: 10.3390/cancers15020504 36672453 PMC9856371

[11] Kitadai R Okuma Y. Treatment strategies for non-small cell lung cancer harboring common and uncommon EGFR mutations: drug sensitivity based on exon classification, and structure-function analysis. Cancers (Basel) (2022) 14(10):219. doi: 10.3390/cancers14102519 35626123 PMC9139782

[12] Wang S Wang Z. Meta-analysis of epidermal growth factor receptor and KRAS gene status between primary and corresponding metastatic tumours of non-small cell lung cancer. Clin Oncol (R Coll Radiol) (2015) 27(1):30–9. doi: 10.1016/j.clon.2014.09.014 25445553

[13] Sherwood J Dearden S Ratcliffe M Walker J. Mutation status concordance between primary lesions and metastatic sites of advanced non-small-cell lung cancer and the impact of mutation testing methodologies: a literature review. J Exp Clin Cancer Res (2015) 34:92. doi: 10.1186/s13046-015-0207-9 26338018 PMC4559261

[14] Zill OA Greene C Sebisanovic D Siew LM Leng J Vu M. Cell-free DNA next-generation sequencing in pancreatobiliary carcinomas. Cancer Discov (2015) 5(10):1040–8. doi: 10.1158/2159-8290.CD-15-0274 PMC4592417 26109333

[15] Meric-Bernstam F Brusco L Shaw K Horombe C Kopetz S Davies MA. Feasibility of Large-scale genomic testing to facilitate enrollment onto genomically matched clinical trials. J Clin Oncol (2015) 33(25):2753–62. doi: 10.1200/JCO.2014.60.4165 PMC4550690 26014291

[16] Corcoran RB Chabner BA. Application of cell-free DNA analysis to cancer treatment. N Engl J Med (2018) 379(18):1754–65. doi: 10.1056/NEJMra1706174 30380390

[17] Paweletz CP Sacher AG Raymond CK Alden RS O'Connell A Mach SL. Bias-corrected targeted next-generation sequencing for rapid, multiplexed detection of actionable alterations in cell-free DNA from advanced lung cancer patients. Clin Cancer Res (2016) 22(4):915–22. doi: 10.1158/1078-0432.CCR-15-1627-T PMC4755822 26459174

[18] Xu S Lou F Wu Y Sun DQ Zhang JB Chen W. Circulating tumor DNA identified by targeted sequencing in advanced-stage non-small cell lung cancer patients. Cancer Lett (2016) 370(2):324–31. doi: 10.1016/j.canlet.2015.11.005 PMC7495502 26582655

[19] Vanni I Coco S Truini A Rusmini M Dal Bello MG Alama A. Next-generation sequencing workflow for NSCLC critical samples using a targeted sequencing approach by ion torrent PGM™ platform. Int J Mol Sci (2015) 16(12):28765–82. doi: 10.3390/ijms161226129 PMC4691076 26633390

[20] Lee CK Kim S Lee JS Lee JE Kim SM Yang IS. Next-generation sequencing reveals novel resistance mechanisms and molecular heterogeneity in EGFR-mutant non-small cell lung cancer with acquired resistance to EGFR-TKIs. Lung Cancer (2017) 113:106–14. doi: 10.1016/j.lungcan.2017.09.005 29110836

[21] Hinrichs JW van Blokland WT Moons MJ Radersma RD Radersma-van Loon JH de Voijs CM. Comparison of next-generation sequencing and mutation-specific platforms in clinical practice. Am J Clin Pathol (2015) 143(4):573–8. doi: 10.1309/AJCP40XETVYAMJPY 25780010

[22] Barnet MB O'Toole S Horvath LG Selinger C Yu B Ng CC. EGFR-Co-Mutated advanced NSCLC and response to EGFR tyrosine kinase inhibitors. J Thorac Oncol (2017) 12(3):585–90. doi: 10.1016/j.jtho.2016.09.001 27639677

[23] Kim EY Cho EN Park HS Hong JY Lim S Youn JP. Compound EGFR mutation is frequently detected with co-mutations of actionable genes and associated with poor clinical outcome in lung adenocarcinoma. Cancer Biol Ther (2016) 17(3):237–45. doi: 10.1080/15384047.2016.1139235 PMC4848002 26785607

[24] Chen JY Cheng YN Han L Wei F Yu WW Zhang XW. Predictive value of K-ras and PIK3CA in non-small cell lung cancer patients treated with EGFR-TKIs: a systemic review and meta-analysis. Cancer Biol Med (2015) 12(2):126–39. doi: 10.7497/j.issn.2095-3941.2015.0021 PMC4493374 26175928

[25] Canale M Petracci E Delmonte A Chiadini E Dazzi C Papi M. Impact of. Clin Cancer Res (2017) 23(9):2195–202. doi: 10.1158/1078-0432.CCR-16-0966 27780855

[26] Labbé C Cabanero M Korpanty GJ Tomasini P Doherty MK Mascaux C. Prognostic and predictive effects of TP53 co-mutation in patients with EGFR-mutated non-small cell lung cancer (NSCLC). Lung Cancer (2017) 111:23–9. doi: 10.1016/j.lungcan.2017.06.014 28838393

[27] Jakobsen JN Santoni-Rugiu E Grauslund M Melchior L Sørensen JB. Concomitant driver mutations in advanced. Oncotarget (2018) 9(40):26195–208. doi: 10.18632/oncotarget.25490 PMC5995236 29899852

[28] Eng J Woo KM Sima CS Plodkowski A Hellmann MD Chaft JE. Impact of concurrent PIK3CA mutations on response to EGFR tyrosine kinase inhibition in EGFR-mutant lung cancers and on prognosis in oncogene-driven lung adenocarcinomas. J Thorac Oncol (2015) 10(12):1713–9. doi: 10.1097/JTO.0000000000000671 PMC4760768 26334752

[29] Yao S Zhi X Wang R Qian K Hu M Zhang Y. Retrospective study of adjuvant icotinib in postoperative lung cancer patients harboring epidermal growth factor receptor mutations. Thorac Cancer (2016) 7(5):543–8. doi: 10.1111/1759-7714.12365 PMC5130296 27766784

[30] Takenaka T Inamasu E Yoshida T Toyokawa G Nosaki K Hirai F. Post-recurrence survival of elderly patients 75 years of age or older with surgically resected non-small cell lung cancer. Surg Today (2016) 46(4):430–6. doi: 10.1007/s00595-015-1200-9 26070907

