# Supplementary material for: Temporal genomic heterogeneity guiding individualized therapy in recurrent non-small cell lung cancer
Source: Front Oncol. 2023 Jul 12;13:1116809. doi: 10.3389/fonc.2023.1116809 (PMC10368968; doi:10.3389/fonc.2023.1116809)
Supplement: Supplementary file 1 [file Table_1.docx]

Supplementary Material

Temporal Genomic Heterogeneity Guiding Individualized Therapy in Recurrent Non-Small Cell Lung Cancer

**Qiyu Fang ^1,2†^, Xiaoying Wan ^2†,^ Angelica D’Aiello^3^, Hui Sun^2^, Weiqing Gu^2^, Yixue Li^5,6^, Caicun Zhou^2^, Boxiong Xie^4^, Qinfang Deng^2^, Haiying Cheng^3*^, and Songwen Zhou^2*^**

^1^ Medical College of Soochow University, Suzhou, Jiangsu Province 215123, China; e-mail@e-mail.com

^2^ Department of Oncology, Shanghai Pulmonary Hospital,Tongji University School of Medicine, Shanghai 200433, China

^3^ Department of Oncology, Montefiore Medical Center / Albert Einstein College of Medicine, Bronx, NY 10461, USA

^4^ Department of Thoracic Surgery, Shanghai Pulmonary Hospital,Tongji University School of Medicine, Shanghai 200433, China

^5^ School of Life Sciences and Biotechnology, Shanghai Jiao Tong University, Shanghai, 200240,China

^6^CAS Key Laboratory for Computational Biology, CAS-MPG Partner Institute for Computational Biology, Shanghai Institute for Biological Sciences, Chinese Academy of Sciences, Shanghai, 200031, China

**^†^** These authors contributed equally to this work

*** First Corresponding Author:**Songwen Zhou
songwenzhou2017@vip.126.com

*** Second Corresponding Author:**Haiying Cheng
hcheng@montefiore.org

**1 Supplementary Table 1 (Gene List)**

| *ABCB1* | *ABL1* | | *ACVR1B* | | *AKT1* | | *AKT2* | | *ALK* | *APC* | |  |  |
| --- | --- | --- | --- | --- | --- | --- | --- | --- | --- | --- | --- | --- | --- |
| *APOB* | *AR* | | *ARAF* | | *ARID1A* | | *ARID2* | | *ATM* | *AXIN1* | |  |  |
| *BAP1* | *BCL2* | | *BRAF* | | *BRCA1* | | *BRCA2* | | *CARD11* | *CBL* | |  |  |
| *CCND1* | *CCND2* | | *CCND3* | | *CCNE1* | | *CD274* | | *CDA* | *CDH1* | |  |  |
| *CDK4* | *CDK6* | | *CDKN2A* | | *CDKN2B* | | *CFTR* | | *CHEK2* | *CREBBP* | |  |  |
| *CRLF2* | | *CSF1R* | | *CTNNB1* | | *CYP19A1* | | *CYP2C8* | | | *CYP2D6* | | *DDR2* |
| *DNMT1* | | *DNMT3A* | | *DPYD* | | *DYNC2H1* | | *EGFR* | | | *EP300* | | *ERBB2* |
| *ERBB3* | | *ERBB4* | | *ERCC1* | | *ERCC2* | | *ESR1* | | | *EZH2* | | *FANCA* |
| *FANCC* | | *FBXW7* | | *FGFR1* | | *FGFR2* | | *FGFR3* | | | *FGFR4* | | *FLT3* |
| *GATA2* | | *GATA3* | | *GNA11* | | *GNAQ* | | *GNAS* | | | *GSTM1* | | *GSTP1* |
| *GSTT1* | | *H3F3A* | | *HRAS* | | *IDH1* | | *IDH2* | | | *IL2RA* | | *IRF5* |
| *JAK1* | | *JAK2* | | *JAK3* | | *KDM6A* | | *KDR* | | | *KEAP1* | | *KIT* |
| *KLF4* | | *KMT2D* | | *KRAS* | | *LRP1B* | | *MAP2K1* | | | *MAP2K2* | | *MAP3K1* |
| *MCL1* | | *MED12* | | *MET* | | *MLH1* | | *MPL* | | | *MS4A1* | | *MSH2* |
| *MSH6* | | *MTHFR* | | *MTOR* | | *MYCN* | | *MYD88* | | | *NEFH* | | *NF1* |
| *NF2* | | *NFE2L2* | | *NOTCH1* | | *NQO1* | | *NRAS* | | | *NTRK1* | | *NUDT15* |
| *PBRM1* | | *PDGFB* | | *PDGFRA* | | *PDYN* | | *PGR* | | | *PIK3CA* | | *PIK3R1* |
| *PMS1* | | *PMS2* | | *PPM1E* | | *PPP2R1A* | | *PTCH1* | | | *PTEN* | | *PTPN11* |
| *RB1* | | *RET* | | *RHOA* | | *ROS1* | | *RRM1* | | | *RUNX1* | | *SF3B1* |
| *SLCO1B1* | | *SMAD4* | | *SMARCA4* | | *SMARCB1* | | *SMO* | | | *SPOP* | | *SPTA1* |
| *STAG2* | | *STK11* | | *TEKT4* | | *TP53* | | *TPMT* | | | *TSC1* | | *TSC2* |
| *TSHR* | | *TYMS* | | *U2AF1* | | *UGT1A1* | | *USH2A* | | | *VHL* | | *WT1* |
| *XPC* | | *XRCC1* | |  | |  | |  | | |  | |  |
